# Exploring the impact of discharged patients’ characteristics on online health information-seeking behaviors: insights from patients’ dilemmas

**DOI:** 10.3389/fpsyg.2025.1500627

**Published:** 2025-02-24

**Authors:** Fei Liu, Xiangyin Kong, Tian Xia, Huijing Guo

**Affiliations:** ^1^School of Economics and Management, Harbin Engineering University, Harbin, China; ^2^School of Management, Harbin Institute of Technology, Harbin, China; ^3^School of Economics and Management, China University of Mining and Technology, Xuzhou, China

**Keywords:** dilemmas, discharged patients, dual-factor model, living with children, online health information-seeking behaviors

## Abstract

**Introduction:**

Aligned with the dual-factor model, this study aims to investigate why discharged patients seek online health information, considering the complexity of patients’ dilemmas. Additionally, we account for specific cultural context factor in China and seek to examine the role of living with children in mitigating the dilemmas faced by discharged patients in their pursuit of additional online information.

**Methods:**

We empirically tested the research model using data collected from 292 discharged patients. The data was examined through structural equation modeling, employing Smart PLS.

**Results:**

The findings suggest that perceived stress facilitates discharged patients’ engagement in seeking online health information, whereas resistance to change and learned helplessness impede such behaviors. Furthermore, our analysis reveals that cohabiting with children moderates the effects of resistance to change on online health information-seeking behavior.

**Discussion:**

In conclusion, this paper extends the literature by examining the role of discharged patients’ characteristics on online health information-seeking behaviors. Following the practices in China, this study involves living arrangements (with children) as an essential factor in the research model. This paper offer suggestions to online providers to make health-related information more suitable for discharged patients.

## 1 Introduction

With the popularization of the Internet and the promotion of online health services, online health information services are becoming increasingly popular among individuals with health information demands (Cline and Haynes, 2001; Gulec et al., 2022). Studies have reported that, on the demand side, approximately 74% of young Americans (Basch et al., 2018) and 65% of Australians (Nikoloudakis et al., 2018) have sought health information online, including conditions, symptoms, and treatment options advice. In China, patients are more accustomed to seeking health resources from traditional offline channels. However, due to the large population in China, it is inconvenient to acquire health information exclusively through offline channels (Matsumoto et al., 2012), leading to patients being unable to satisfy their quest for health information. According to Xinhua Net, the news website of the Chinese government, online health information-seeking behaviors rank as the second main category of people’s information-seeking behaviors. With the development of the Chinese Internet in the healthcare sector, more and more people are seeking web-based health information and services (Cao et al., 2016; Zhang et al., 2017).

Health information-seeking behaviors involve acquiring health-related information to assist individual medical decision-making and self-management (Lambert and Loiselle, 2007). Online health information services can benefit healthy people by preventing disease (Zhao et al., 2021). It can also help patients manage their health conditions. Patients who suffer from diseases can search for health-related information to find ways to minimize their suffering related to health conditions, such as recovery problems. Online information could also provide an alternative explanation of doctors’ suggestions for patients (Chang and Huang, 2020).

Patients usually have health information demands during the complex self-management period (Sedrak et al., 2020). Although online health information services provide a more convenient and low-cost way for patients to meet their information needs, patients still cannot avoid the dilemmas discussed herein. In China, discharged patients lack basic health-related information, and they cannot acquire well-matched information or distinguish it from less relevant information (Meng et al., 2019). In addition to technical factors, online-seeking behaviors are influenced by the characteristics of discharged patients themselves. Although searching for health information online has become common among patients, existing research primarily focuses on the health information seek for the general patient population (Kim et al., 2023). It is important to note that discharged patients, particularly in China, are often middle-aged or elderly individuals who have unique experiences and may face additional challenges when seeking health information online.

In this context, studies are needed to understand the real dilemmas discharged patients face when seeking online health information. Such dilemmas include dependence on clinical opinions (Eibergen et al., 2018), vulnerability (Walter et al., 2019), extensive negative emotions (Yan et al., 2021), and stress (Musarezaie et al., 2019) after a long clinical experience. They usually experience a high level of perceived stress while dealing with their own health conditions (Liu et al., 2021) and are unwilling to change their original information acquisition routes (Shahbaz et al., 2020). At the same time, they usually feel a helplessness when seeking health information online due to the conflicting information they find (Fassett-Carman et al., 2019; Wanke and Schwabe, 2020).

Resistance is often manifested as the failure of users to transition from existing business models to new ones (Polites and Karahanna, 2012). Online seeking health information provide a new source of information that is accessible 7 days a week and without spatial limitations. Moreover, this method has indeed supplanted traditional offline means of obtaining health information to some extent. Unlike typical patients, discharged patients may encounter greater resistance when shifting to online sources for health information, as they have become accustomed to receiving it from professional healthcare providers in person. This resistance to seeking online health information among discharged patients may be related to their hospitalization experiences. However, existing studies on the impact of patient characteristics on online health information-seeking behaviors have primarily focused on facilitating factors, such as the roles of health-related stress (de Looper et al., 2021) and health literacy (Nangsangna and Vroom, 2019). There has been limited attention to the influence of discharged patients’ characteristics on their online health information-seeking behaviors, particularly concerning inhibitory factors. Understanding these factors not only enables health information providers to develop effective dissemination strategies but also enhances our comprehension of facilitating factors from a new perspective (Tsai et al., 2019). Drawing on the dilemmas discharged patients face, this study adopted the dual-factor model to understand the role of discharged patients’ characteristics in their information-seeking processes. This study analyzed how facilitating and inhibitory factors, especially the latter, affect online health information-seeking behaviors. Thus, the first research question is as follows:

*RQ 1*. Which factors, especially inhibitors, can impact patients seeking health information online based on their dilemmas after discharge?

At the same time, considering the traditional Chinese culture, the role the family plays in health information acquisition and decision-making cannot be negligible (Zhang et al., 2020). To assist with care delivery and avoid accidents, discharged patients usually live with their children, if they have any, making living arrangements essential for patients’ self-management (Irani et al., 2019). Thus, the second research question is as follows:

*RQ 2*. How do living arrangements affect the relationship between discharged patients’ characteristics and online health information-seeking behaviors?

To narrow this research gap, we adopted the dual-factor model and proposed a research model to investigate the enablers and inhibitors of patients’ online health information-seeking behaviors. According to the dilemmas discharged patients face, we regard the enablers (perceived stress) and inhibitors (resistance to change and learned helplessness) as factors affecting online health information-seeking behaviors.

We tested our hypotheses using a survey of 292 respondents. The results supported all the direct influencing routes. Perceived stress was positively associated with online health information-seeking behaviors, while resistance to change and learned helplessness were negatively associated with these behaviors. By comparing patients who live with their children with those who do not, we found that living with children can only significantly weaken the relationship between resistance to change and online health information-seeking behaviors. The result rejected the moderating effect of the other two direct routes.

This paper contributes to the health-related online-seeking literature in several ways. First, this paper extended the literature by examining the positive relationship between the perceived stress of discharged patients and their online health information-seeking behaviors. Second, we enriched the literature on online health information-seeking behaviors by introducing the role of inhibitors following the health and information dilemmas discharged patients face. Third, this study contributes to understanding how living arrangements (i.e., with children) influence discharged patients’ online health information-seeking behaviors. In practical terms, this research starts from the dilemma discharged patients face to better understand why patients do or do not use online information-seeking services. Using the findings, this paper offers suggestions to online health information providers to make online information more relevant to discharged patients. Finally, this paper emphasizes the important role of living arrangements on discharged patients’ online health information-seeking behaviors.

The rest of the paper proceeds as follows. In the theoretical background section, we describe prior research on online health information-seeking behaviors, the dual-factor model, perceived stress, resistance to change, and learned helplessness to set the literary context for our study, and the research model incorporates the dual-factor model. Next, the research methodology in the subsequent section, including data collection and analysis, to empirically test our hypothesized models and introduces the statistics and calculation results of the partial least squares. Finally, this study provides the main findings, implications, and limitations.

### 1.1 Online health information-seeking behaviors

Online health information-seeking behaviors refers to the process by which individuals obtain health information about diseases, treatment plans, and prognoses through search engines (e.g., Google and Baidu), dedicated online health platforms (e.g., Haodaifu), and social media platforms (e.g., Facebook and WeChat) (Chen et al., 2025). As popular internet behaviors, online health information-seeking behaviors may generate multiple health-related outcomes, such as assisting patients’ self-diagnosis under specific health conditions (Jiang and Street, 2017; McMullan et al., 2019) and supporting health or medical services distribution (Shamlou et al., 2022). Driven by multiple motives, needs, and environmental factors, people may adopt online health information services (Reifegerste et al., 2019). Related phenomena have led researchers to explore the influencing factors of online health information-seeking behaviors in recent years.

Previous studies usually examined the impact of internal (personal traits) and external (environmental characteristics) factors on online information-seeking behaviors. Such studies found that individual health-related characteristics, such as lacking health literacy (Wong and Cheung, 2019) and suffering from disease symptoms (Berle et al., 2020), were the essential influencing factors of online health information-seeking behaviors. At the same time, social surroundings, such as lacking health resources (Xiao et al., 2014), could also influence online health information-seeking behaviors.

Patients are key consumption populations adopting online health information services (Sedrak et al., 2020). To date, a rich and extensive literature has examined the enablers of patients’ online health information-seeking behaviors (de Looper et al., 2021; Nangsangna and Vroom, 2019; Zhao et al., 2020). As a part of healthcare services, online health information could compensate for the shortage of traditional medical services and expand patients’ medical resources (Miller and Bell, 2012; Zhao et al., 2020). Then, with the expectation of improving personal health status and self-management levels, patients may seek information online (Kitchens et al., 2014). Yet once patients are discharged from hospitals, they may face more obstacles when seeking online health information due to their unique characteristics.

### 1.2 Dual-factor model of online health information-seeking behaviors

The dual-factor model highlights the need to consider both enablers and inhibitors when using information systems (Cenfetelli, 2004). Enablers are those factors that promote system usage whereas inhibitors focus on the factors that predict users’ tendency to reject the technology. According to the dual-factor model, it is essential to understand the inhibitors from the perspective of personal characteristics, such as technology anxiety (Guo et al., 2013) and dispositional resistance to change (Hsieh, 2016), when using information systems. Focusing on the elderly, previous studies have investigated the correlation of online health information-seeking behaviors with possible barriers, such as low e-health literacy, conflicting information, and web design factors (Pourrazavi et al., 2022). Due to past disease experiences and uncontrollable health conditions, discharged patients may generate negative emotions and dependence on clinical opinions when they encounter barriers while seeking health information, leading to a negative impact on factors related to use behaviors (Bhattacherjee and Hikmet, 2007). Although the existing literature has studied online health information-seeking behaviors from many aspects, it is rare to treat discharged patients as subjects while exploring the inhibitors of these demand groups. Hence, in this study, we adopted the dual-factor model to understand the inhibitors of discharged patients’ online health information-seeking behaviors.

### 1.3 Dilemmas and characteristics of discharged patients

Patients usually differ from general population in their specific health concerns and stressful situations. Due to the uncertainty and perceived risks of their health status, seeking online health information is more purposeful for patients (Rains and Tukachinsky, 2015), such as seeking disease treatment information, than for general populations. Researchers have found that primarily personal factors, such as stress and anxiety generated from complex health conditions (McMullan et al., 2019), drive patients to seek health information online. Due to long-term clinical experience, discharged patients tend to be dependent on clinical opinions (Eibergen et al., 2018), vulnerable (Walter et al., 2019), full of negative emotions (Yan et al., 2021), and stressed (Musarezaie et al., 2019). The dilemma discharged patients face, caused by their complicated conditions, recovery problems, distance from offline medical resources, and special needs for disease-related information, will affect their online health information-seeking behaviors. Thus, representative personal characteristics of discharged patients were identified based on several specific dilemmas.

Unlike general populations who are remote from health resources, discharged patients have usually adopted several clinical resources to deal with their health problems during hospitalization. Although patients receive clinical guidance and advice, limited health resources support them in going through the uncertainty and unprovability of health conditions after discharge (Chang and Huang, 2020). These health-related conditions may generate related concerns and contribute to a higher level of perceived stress for discharged patients than general individuals. Perceived stress reflects the subjective evaluation of the stress level individuals experience when they encounter a stressful objective event (Cohen et al., 1983). Patient populations usually have problems with perceived stress (Liu et al., 2021). Related studies have shown that patients experience higher levels of perceived stress due to health threats (Liu et al., 2021) and emotional distress (Yan et al., 2021) than healthy people. One previous study demonstrated that patients with stressful health conditions and concerns have a greater need for health information, leading to frequent health information-seeking behaviors (Musarezaie et al., 2019). When they are discharged from the hospitals, perceived stress becomes a significant characteristic of discharged patients in health information acquisition to be explored.

Previous studies have indicated that, after discharge, patients tend to search for information using health queries to reduce uncertainty (Yan et al., 2021). As an effective strategy to relieve health-related stress, online health information-seeking behaviors, which are low-cost ways to acquire health-related information, can comfort stressed discharged patients. Thus, we hypothesize that:

*Hypothesis 1*. Discharged patients’ perceived stress increases the number of their online health information-seeking behaviors.

As health-related problems have accompanied discharged patients for some time, they are usually dependent on clinical opinions and a resistance to changing their information behaviors. Resistance to change refers to the unwillingness to take action to adapt to the pressure to change the status quo (Shahbaz et al., 2020). Previous studies have found that resistance to change could negatively impact an individual’s intention to change health behaviors (Deng et al., 2014; Laumer et al., 2016). Resistance to change also weakens the positive relationship between intention to use and actual use (Shahbaz et al., 2020). Discharged patients with long-term illness experience have become accustomed to seeking clinical support from offline physicians (Sedrak et al., 2020), causing them to distrust alternative health-related resources. As a result, discharged patients may resist changing their health behaviors due to the influence of the external environment. Once discharged patients who are used to offline diagnosis and treatment opinions will exhibit resistance to change as a key characteristic of their online health information seeking.

Online health information services could provide an accessible way for discharged patients to find health-related information (Soroya et al., 2021). Discharged patients’ efforts to seek health-related information online could reshape their ways of handling health issues (Guo et al., 2013). However, discharged patients are mainly accustomed to adopting information acquired from face-to-face hospital medical services rather than using online information technology, which could cause them to resist changing their previous health habits (Deng et al., 2014) related to acquiring health information. In sum, we believe that previous health information-acquiring habits may generate a resistant attitude that leads to decreased online health information-seeking behaviors of patients after discharge. Thus, we hypothesize that:

*Hypothesis 2*. Discharged patients’ resistance to change decreases the number of their online health information-seeking behaviors.

Online health information-seeking behaviors may generate unpredictability and uncertainty in the search results (Soroya et al., 2021), which discharged patients who lack basic health-related knowledge may not be able to discern (Meng et al., 2019). And individual discharged patients have complicated health issues (Musial et al., 2020), while online health information services usually lack personalization (Meng et al., 2019), resulting in difficulties in acquiring well-matched information online. This dilemma may result in helplessness for discharged patients seeking information online (Fassett-Carman et al., 2019; Wanke and Schwabe, 2020). Learned helplessness is grounded in cognitive learning theory (Maier, 1980) and indicates that people think, feel, or act passively and helplessly when they cannot control their surroundings (Trindade et al., 2020). The root of learned helplessness is the belief that the individual has lost control of the situation and is incapable of making changes (Baum et al., 1986). Because of the uncertainty of clinical treatment (Djulbegovic et al., 2011), discharged patients usually feel incapable of dealing with their health conditions through personal efforts, resulting in learned helplessness. Previous studies have shown that learned helplessness harms mental health and behavioral changes (López Steinmetz et al., 2021; Xue et al., 2023). If patients’ online efforts to seek health information are unable to improve their health outcomes, the characteristics of learned helplessness will become more pronounced.

If individuals believe that their efforts may not produce improved health conditions and may even worsen these conditions, they may stop their attempts to find health information (Xue et al., 2023; Ying et al., 2021) due to their helplessness. Feelings of helplessness when repeatedly seeking health information online without changing the patient’s health conditions lead to the emergence of learned helplessness (Benko et al., 2021), resulting in a negative attitude toward seeking online health information. Thus, we hypothesize that:

*Hypothesis 3*. Discharged patients’ learned helplessness decreases the number of their online health information-seeking behaviors.

### 1.4 Living arrangement

People who live with their children tend to have improved health cognition (Yu et al., 2022) and more appropriate health management behaviors. Understanding the role of this living arrangement in discharged patients’ online health information-seeking behaviors is essential.

Although seeking information online is an essential strategy for releasing perceived stress, discharged patients living with their children have other strategies for coping with stress issues, such as face-to-face communication with their children (Yan et al., 2021). We can conclude that, under the same level of perceived stress, discharged patients who live with their children may find more alternative strategies to relieve perceived stress than those who do not live with their children. Therefore, we hypothesize that:

*Hypothesis 4*. Under the same level of perceived stress, discharged patients who live with their children seek less online health information than discharged patients who do not live with their children.

Related studies have demonstrated that social support (such as the companionship of children) is associated with attitude change (Stroebe and Diehl, 1988). Living with their children may reduce discharged patients’ online health information-seeking behaviors caused by a resistant attitude and may enhance their self-management behaviors (Irani et al., 2019). Likewise, discharged patients who live with their children could also reduce online health information-seeking behaviors caused by resistance to change through social support provided by their children. Then, we hypothesize that:

*Hypothesis 5*. Under the same level of resistance to change, discharged patients who live with their children seek more online health information than discharged patients who do not live with their children.

People who live with their children usually have less leisure time and are busy dealing with various domestic affairs (Walter and Haun, 2021), making them more vulnerable to managing situations with little outside resources. As a result, people who live with their children may experience weakened positive thinking (Walter and Haun, 2021). For discharged patients with feelings of learned helplessness, the negative thinking may be enhanced by living with their children. Therefore, we hypothesize that:

*Hypothesis 6*. Under the same level of learned helplessness, discharged patients who live with their children seek less online health information than discharged patients who do not live with their children.

### 1.5 Research model

**Figure 1:**
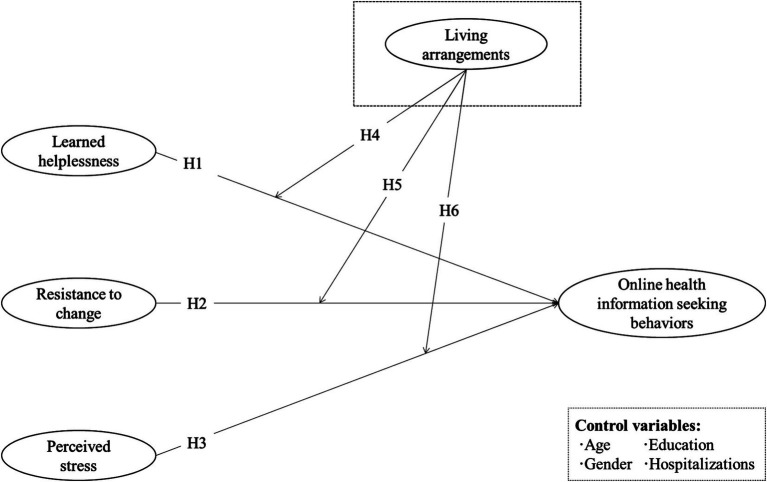
Research model.

Following the dual-factor model, we developed a research model to understand the enabler (perceived stress) and inhibitors (resistance to change and learned helplessness) in discharged patients’ online health information-seeking behaviors (see Figure 1). We built this research model from the insights of discharged patients’ dilemmas related to their health conditions and online information service engagement. Furthermore, considering the essential role of living arrangements in discharged patients’ daily life, we proposed that living with children is a moderator that affects the relationship among our three direct affect routes.

We also included several personal characteristics in the model as control variables to alleviate the influence due to covariance issues. Previous literature has suggested that individuals’ age, gender (Weber et al., 2020), levels of education (Berle et al., 2020), and disease experience (Sedrak et al., 2020) can influence their health-seeking behaviors on the internet. Based on our research context, we adopted the number of hospitalization experiences instead of the disease experience. We thus controlled the effects of these factors on online health information-seeking behaviors.

## 2 Methodology

### 2.1 Participants

To pretest the questionnaire, we initially collected data from a small sample of hospitalized patients in offline hospitals. However, as we began to collect the questionnaires, the spread of COVID-19 resulted in the loss of opportunities for face-to-face interactions with patients. Therefore, we utilized the data collection service of China’s leading online survey platform, Wenjuanxing, to manage the survey. There were two primary reasons for conducting the survey online. First, it relates to our research topic, online health information seeking behaviors, which is inherently digital and primarily targets internet users. Second, it has been reported that the internet has become the preferred source of information for patients, surpassing healthcare professionals (Chen et al., 2025). Thus, the online survey can be considered representative to some extent. The questionnaire was divided into two parts. In the first part, respondents were informed about the purpose of data collection and provided their consent. Subsequently, they answered questions regarding their hospitalization experience and health management after discharge. In the second part, they were asked about their perspectives on online health information seeking behaviors during their self-management period post-discharge. To ensure data quality and reduce social desirability bias, we have taken several measures when designing questionnaires and cleaning up the collected data. First, we added attention traps and reverse coding questions to reduce single-method bias. Second, we ruled out all cases where the problem is missing a value or a similar value. The questionnaires were distributed online from September 1 through September 30, 2021.

The website initially collected 472 questionnaires, however, due to concerns about patient privacy and the absence of effective incentives, 158 responses were incomplete. Of the 314 pieces of data the website provided, the authors excluded another 22 pieces of data with more than eight consecutive measurement items of the same value (indicating that the respondents did not answer seriously). Ultimately, 292 valid questionnaires were used to conduct the quantitative analysis.

Table 1 presents the demographic characteristics. According to the data, the proportion of women in the survey was a bit higher than that of men, reaching 54.46%, and the age was mainly 25–34 and 35–44 years old. Most of the survey group had a master’s degree or above, and they had been hospitalized once or twice. Meanwhile, most of them did not live alone; 59.25% lived with their children.

**Table 1:** Demographic characteristics.

	Frequency	Percentage (%)
Gender		
Male	133	45.54
Female	159	54.46
Age		
18–24	17	5.82
25–34	102	34.93
35–44	89	30.48
45–54	63	21.58
55–64	13	4.45
≥65	8	2.74
Education		
Middle school and below	9	3.08
Technical secondary school and high school	29	9.93
Junior college	38	13.01
Bachelor’s degree or above	216	73.98
Hospitalization frequency		
0	18	7.00
1	161	54.14
2	83	28.98
≥3	30	9.87
Live alone		
Yes	18	6.16
No	274	93.84
Live with children		
Yes	173	59.25
No	119	40.75

### 2.2 Measures

To test the variables in this study, we adopted and developed the measurement items from previous studies. We first adopted the measurement of online health information-seeking behaviors (Bell et al., 2011) and then developed the measurement items based on the practices in China. We summed the five answers up to measure the number of online health information-seeking behaviors. We also adopted measurements of learned helplessness (Quinless and Nelson, 1988), resistance to change (Bhattacherjee and Hikmet, 2007), and perceived stress (Cohen et al., 1983) from previous research on a 7-point Likert-type scale ranging from 1 (“strongly disagree”) to 7 (“strongly agree”). We examined living with children by directly asking the participants whether they lived with their children (regardless of whether they have a child or not). Based on the above support and the background of this study, we translated the questionnaire into Chinese and made it more suitable for answering.

### 2.3 Data analysis

Partial least squares (PLS), a form of structural equation modeling (SEM), provides value for behavioral research fields (Lowry and Gaskin, 2014). PLS offers some data analysis advantages, and we used this technique to test the research model for the following reasons. First, PLS has low requirements on the measurement scale and requires a relatively small sample size (Hair et al., 2011). Moreover, SEM allows the simultaneous operation of multiple related equations, and a model is measured multiple times. PLS can be used for both the confirmation of existing model parameters and exploratory verification (Chin, 1998).

We used the Smart PLS 4.0 software as the statistical tool to examine the research model. We analyzed our data in three stages. The first stage was testing the measurement model, which evaluated the reliability and validity of the construct to ensure its appropriateness. In the second stage, we tested the structural model and the hypotheses. Finally, we used a multigroup analysis to compare whether the respondents live with their children or not.

## 3 Results

### 3.1 Measurement model testing

We evaluated model reliability by examining composite reliability (CR) and average variance extracted (AVE) (Fornell and Larcker, 1981; Hsu and Lin, 2008). The results are shown in Table 2. The CR ranged was from 0.864 to 0.937 (higher than 0.7), and the AVE ranged from 0.672 to 0.789 (higher than 0.5). Comparing these two sets of results with the critical values of 0.7 and 0.5, respectively (Fornell and Larcker, 1981; Hsu and Lin, 2008; Chin, 1998) indicated that the structure had very good reliability. Discriminant validity could be measured by the square root of AVE (Fornell and Larcker, 1981). As shown in Table 2, the square root of AVE ranged from 0.820 to 0.888, which was greater than variable correlations. Thus, it could be concluded that the discriminant validity was acceptable.

**Table 2:** Reliability and discriminant validity.

Constructs	Cronbach’s α	CR	AVE	LH	PS	RTC
LH	0.706	0.864	0.762	**0.873**		
PS	0.837	0.891	0.672	0.233	**0.820**	
RTC	0.915	0.937	0.789	0.178	0.293	**0.888**

The bold diagonal data refer to the square roots of AVE.

We evaluated the convergent validity by measuring the items’ loadings. The results in Table 3 show that the loadings of all items ranged from 0.782 to 1.000, all of which were greater than 0.70 (Anderson and Gerbing, 1988). In addition, the loadings of these items were greater than their cross-loadings with other items (Gefen and Straub, 2005), indicating that the structure had good convergent validity.

**Table 3:** Convergent validity.

	Item loading	OHIB	LH	RTC	PS
OHIB	1.000	**1.000**	−0.127	−0.136	0.220
LH1	0.937	−0.102	**0.937**	0.143	0.261
LH2	0.804	−0.132	**0.804**	0.184	0.115
RTC1	0.832	−0.062	0.157	**0.832**	0.340
RTC2	0.901	−0.109	0.155	**0.901**	0.291
RTC3	0.904	−0.122	0.206	**0.904**	0.273
RTC4	0.914	−0.155	0.129	**0.914**	0.205
PS1	0.834	0.213	0.156	0.241	**0.834**
PS2	0.836	0.160	0.192	0.326	**0.836**
PS3	0.782	0.161	0.204	0.169	**0.782**
PS4	0.826	0.185	0.211	0.230	**0.826**

The bold data refer to the factor loadings of each construct.

### 3.2 Structural model testing

Based on our research hypotheses, the measurement of the structural model was tested in stage 1. The PLS results of the structural model are shown in Table 4. We tested the effects of learned helplessness, perceived stress, resistance to change, and control variables (age, hospitalization frequency, education, and gender) on online health information-seeking behaviors.

**Table 4:** Partial least squares results.

Path	Coefficient (bi)	t-statistics	R Square
LH → OHIB	−0.144	2.635***	0.141
RTC → OHIB	−0.195	2.798***	
PS → OHIB	0.273	4.300***	
AGE → OHIB	0.079	1.216	
HF → OHIB	0.103	1.694	
EDU → OHIB	−0.041	0.672	
GEN → OHIB	0.105	1.060	

*p < 0.1; **p < 0.05; ***p < 0.01.

The results showed that learned helplessness and resistance to change were negatively associated with online search behavior (b1 = −0.144, *t* = 2.635; b2 = −0.195, *t* = 2.798) while perceived stress was positively associated with online search behavior (b3 = 0.273, *t* = 4.300), thereby lending support to H1, H2, and H3. Finally, the control variables had insignificant effects on online health information-seeking behaviors (b4 = 0.079, *t* = 1.216; b5 = 0.103, *t* = 1.694; b6 = −0.041, *t* = 0.672; b7 = 0.105, *t* = 1.060).

Our low R-squared values can be explained by three aspects. First, due to the difficulty in predicting individual behavior, low values are common in social science research that predicts human behavior (online health information seeking) and widely exist in previous studies (Agarwal and Prasad, 1999). Second, our study starts from the dilemma discharged patients face in order to investigate their online health information-seeking behaviors, which is inherently complex, so a lower R-squared value is also acceptable. Finally, there are significant differences in the factors that affect the online health information-seeking behaviors of discharged patients, and we did not consider them based solely on individuals’ predicament.

### 3.3 Multigroup analysis

Multigroup analysis (MGA) is an efficient approach to evaluate moderation using partial least squares path modeling (PLSPM), as recent research has shown (Cheah et al., 2020). We tested the hypotheses by comparing whether individuals lived with their children. Following Keil et al. (2000), we statistically compared the corresponding path coefficients and computed the *p*-value (see Table 5). The *p*-value suggests that H5 is supported, but H4 and H6 are not.

**Table 5:** Multigroup analysis results.

Path	Path coefficients-difference	*p* value
LH → OHIB	0.004	0.975
RTC → OHIB	0.284	0.017**
PS → OHIB	0.047	0.689

*p < 0.1; **p < 0.05; ***p < 0.01.

## 4 Discussion

The overall goal of this study was to explore the factors influencing online health information-seeking behaviors, especially the inhibitors, rooted in the dilemmas of discharged patients. We presented a theoretical model based on the dual-factor model, testing the positive (perceived stress) and negative (learned helplessness and resistance to change) impact on online health information-seeking behaviors. As living with children can alter discharged patients’ health-management behaviors (Yu et al., 2022), such as information-related behaviors (Zhou et al., 2019), we examined living with children as a moderating variable of the direct routes.

### 4.1 Discussion of findings

The results supported all the directed influencing relationships on online health information-seeking behaviors. However, only the moderating role of living with children in the relationship between resistance to change and online health information-seeking behaviors was supported; the other two moderating hypotheses were rejected by the results.

First, the results demonstrate that perceived stress significantly positively affects the number of online health information-seeking behaviors. When facing the same dilemma, discharged patients with a higher level of perceived stress seek more online health information than patients with a lower level of perceived stress. As an effective and low-cost strategy to relieve health-related stress, online health information-seeking behaviors can comfort stressed discharged patients.

Second, resistance to change and learned helplessness significantly negatively impact the number of online health information-seeking behaviors. As expected, the relationship between resistance to change and online health information-seeking behaviors is significantly negative, suggesting that discharged patients with resistance to change may rely on existing information-accessing habits instead of seeking information online. Meanwhile, with a negative attitude of learned helplessness toward online health information, discharged patients decrease the number of online health information-seeking behaviors.

Third, the results confirmed that living with children moderates the relationship between resistance to change and online health information-seeking behaviors. Discharged patients living with children demonstrate fewer online health information-seeking behaviors caused by resistance to change than patients who do not live with their children. The other two moderation effects of living with children on direct relationships are insignificant. The following considerations may explain these phenomena. Living with children is an essential social determinant of health-related behaviors, with a high dependence on age (Rattay and von der Lippe, 2020). In this study, our data sample mainly consisted of patients in the age groups of 25–34 and 35–44, which may have resulted in the insignificant moderating effect of living with children. In addition, although people who live with their children may weaken their positive thinking (Walter and Haun, 2021), a related paper also concluded that parents tend to show positive attitudes toward media (Vittrup et al., 2016), resulting in a less clear role of living with children in online health-information seeking behaviors.

### 4.2 Theoretical and practical implications

Following dual-factor model, we conduct a theoretical model of influencing online health information-seeking behaviors, especially inhibitors. This study contributed to the existing literature in several ways.

First, we extended the literature by examining the positive relationship between discharged patients’ perceived stress and their online health information-seeking behaviors. For the general population, research has already provided support for the claim that perceived stress has an effect on search behaviors (Kugbey et al., 2019; Liu et al., 2021). In our study, we extended the influence to discharged patients based on their health dilemmas.

Second, to give full insights into discharged patients’ online information-seeking behaviors, we explored the inhibitors of online health information-seeking behaviors. Following the dilemma of discharged patients’ contradiction in previous health information-adoption habits and online-seeking approach, we empirically proved that resistance to change could be an inhibitor of online information-seeking behaviors. Furthermore, as discharged patients experience feelings of helplessness about online seeking results, we found learned helplessness could decrease the number of online health information-seeking behaviors.

Third, this study contributes to understanding how living with children influences discharged patients’ online health information-seeking behaviors. This study provides a new viewpoint on the moderating effect between resistance to change and online health information-seeking behaviors.

This research also offers practical implications in assisting discharged patients in coping with dilemmas for three groups—namely, discharged patients themselves, online health information service providers, and family members.

First, our research analyzed the real dilemma of discharged patients, offering insights into understanding the reasons why they cannot escape these dilemmas using the available low-cost online health information services. Although discharged patients have limited health resources from traditional medical systems, they might not search for alternative online health-related information services due to their previous information adoption habits and feelings of helplessness related to online seeking results.

Second, this study’s results lead to suggestions for online health information service providers to adjust the online content and information delivery ways to achieve available and appropriate services to assist discharged patients. These providers could link online services with traditional hospitals to reduce patients’ resistance to online health information service adoption. At the same time, the provider of online health information should enhance the credibility and personality of such information to reduce feelings of learned helplessness. By following these suggestions, some of the inhibitors of online health information-seeking behaviors can be removed.

Third, living arrangements could provide an essential role for discharged patients to seek health information online. This study found that discharged patients who live with their children might have a weakened relationship between discharged patients’ characteristics and online health information-seeking behaviors, illustrating the critical role of living with children in changing the online-seeking behaviors generated by a resistant attitude.

### 4.3 Limitations and potential for future study

Despite this study’s theoretical and practical implications, we should also address several limitations of the study. First, our study used online survey data to test the facilitators and inhibitors of online health information-seeking behaviors for discharged patients. The reason for collecting data online was that the cooperating hospital could not support survey distribution during the COVID-19 pandemic in China. The research sample was mainly between the ages of 25–34 and 35–44. However, older adults are an essential part of discharged patients. In our future research, we will explore the inhibitors of online health information-seeking behaviors among older adult patients after discharge.

Second, the research sample of this study was patients with multiple health conditions who had been hospitalized and discharged. However, different diseases may result in different health conditions and self-management behaviors for discharged patients. In our future research, we will analyze and examine discharged patients with specific diseases, such as chronic diseases, to achieve more detailed insights into these disease characteristics.

## Data Availability

The original contributions presented in the study are included in the article/supplementary material, further inquiries can be directed to the corresponding author.
